# Dietary Polyphenols in Brain Aging: Molecular Mechanisms and Implications for Neurodegeneration

**DOI:** 10.3390/nu18091470

**Published:** 2026-05-05

**Authors:** Noémi Mózes, János Tamás Varga, Dominik Szwajgier, Agata Kryczyk-Poprawa, Virág Zábó, Andrea Lehoczki, Ágnes Lipécz, Tamás Csípő, Vince Fazekas-Pongor, Dávid Major, Péter Varga, Attila Matiscsák, Mónika Fekete

**Affiliations:** 1 Institute of Preventive Medicine and Public Health, Faculty of Medicine, Semmelweis University, 1085 Budapest, Hungary; mozes.noemi@semmelweis.hu (N.M.); zabo.virag@semmelweis.hu (V.Z.); ceglediandi@freemail.hu (A.L.); lipecz.agnes@semmelweis.hu (Á.L.); csipo.tamas@semmelweis.hu (T.C.); pongor.vince@semmelweis.hu (V.F.-P.); major.david@semmelweis.hu (D.M.); varga.peter@semmelweis.hu (P.V.); 2 Health Sciences Division, Doctoral College, Semmelweis University, 1085 Budapest, Hungary; 3 Fodor Center for Prevention and Healthy Aging, Semmelweis University, 1085 Budapest, Hungary; 4 Department of Pulmonology, Semmelweis University, 1083 Budapest, Hungary; varga.janos.tamas@semmelweis.hu; 5 Department of Biotechnology, Microbiology and Human Nutrition, University of Life Sciences in Lublin, 20-704 Lublin, Poland; dominik.szwajgier@up.edu.pl; 6 Department of Inorganic Chemistry and Pharmaceutical Analytics, Faculty of Pharmacy, Jagiellonian University Medical College, 31-008 Krakow, Poland; agata.kryczyk@uj.edu.pl; 7 S-CAPE Cognitive and Health Prevention Research Group, Faculty of Health Sciences, Semmelweis University, 1088 Budapest, Hungary; matiscsak.attila@semmelweis.hu; 8 Department of Social Sciences, Faculty of Health Sciences, Semmelweis University, 1085 Budapest, Hungary

**Keywords:** dietary polyphenols, brain aging, neurodegeneration, cognitive decline, oxidative stress, neuroinflammation, gut–brain axis, Mediterranean diet, precision nutrition

## Abstract

Background/Objectives: Population aging is accompanied by a rapidly increasing burden of neurodegenerative disorders, particularly Alzheimer’s and Parkinson’s diseases. Within the geroscience framework, targeting fundamental mechanisms of aging may delay the onset or progression of multiple age-related conditions. Dietary factors, especially plant-derived polyphenols, have gained increasing attention due to their potential to modulate molecular pathways involved in brain aging. This narrative review aims to integrate current evidence on dietary polyphenols and their role in modulating the molecular mechanisms underlying brain aging and neurodegeneration. Methods: This narrative review synthesizes findings from molecular, experimental, epidemiological, and clinical studies to provide an integrated assessment of the effects of dietary polyphenols on key cellular pathways involved in brain aging. Results: Polyphenols are widely present in plant-based foods, and polyphenol-rich dietary patterns—particularly the Mediterranean and MIND diets—have been consistently associated in observational studies with a reduced risk of cognitive decline. Mechanistic evidence, derived predominantly from in vitro and animal studies with limited validation in humans, suggests that polyphenols may influence key hallmarks of aging, including oxidative stress, chronic inflammation, mitochondrial dysfunction, cellular senescence, and impaired proteostasis. These effects are mediated through pathways such as Nrf2, NF-κB, AMPK, mTOR, and SIRT1, as well as via gut–brain axis interactions. However, clinical evidence remains heterogeneous. Conclusions: Dietary polyphenols represent a biologically plausible and promising, yet not fully validated, nutritional strategy for promoting healthy brain aging. Their translation into clinical practice is limited by low bioavailability, substantial interindividual variability, and the lack of large-scale, long-term randomized controlled trials.

## 1. Introduction

Aging is the strongest known risk factor for most chronic diseases, particularly neurodegenerative disorders [1]. Ongoing demographic transitions have led to a rapidly aging global population, accompanied by a substantial increase in the prevalence of dementia and related neurological conditions [2]. The incidence of Alzheimer’s disease (AD), Parkinson’s disease (PD), and other neurodegenerative disorders is projected to rise markedly in the coming decades, placing a growing burden on healthcare systems and society [3]. Increasing evidence suggests that these conditions are not isolated disease entities but are closely linked to the progressive dysregulation of fundamental biological processes associated with aging [4].

Within the geroscience framework, aging-related mechanisms—collectively referred to as the key hallmarks of aging (e.g., oxidative stress, inflammation, and mitochondrial dysfunction)—are considered shared drivers of multiple age-related diseases [5,6]. Modulation of these pathways may therefore influence the onset and progression of several chronic conditions simultaneously [7]. The central nervous system (CNS) is particularly vulnerable to these processes due to the high metabolic demand and limited regenerative capacity of neurons [8]. Brain aging is characterized by the progressive dysregulation of these interconnected processes, including mitochondrial dysfunction, neuroinflammation, blood–brain barrier (BBB) impairment, and accumulation of misfolded proteins, including amyloid-β and α-synuclein [9,10].

Given the complexity of aging biology, increasing attention has focused on modifiable lifestyle factors that can influence these molecular pathways [5]. Among these, nutrition represents a key determinant of healthy aging and chronic disease prevention [11,12,13,14]. Dietary patterns influence not only nutrient supply but also cellular metabolism, redox balance, inflammatory signaling, and immune function [12,15,16]. In this context, bioactive compounds present in foods have emerged as important modulators of aging-related pathways [14,17,18,19,20,21,22].

Polyphenols are among the most extensively studied plant-derived bioactive compounds [23]. They are widely distributed in foods such as fruits, vegetables, tea, cocoa, coffee, whole grains, and extra-virgin olive oil [24], and include diverse subclasses such as flavonoids, phenolic acids, stilbenes, and lignans [25]. These compounds exert pleiotropic biological effects, including antioxidant, anti-inflammatory, and metabolic regulatory actions, as well as modulation of cellular signaling pathways primarily demonstrated in experimental systems [26].

Epidemiological and clinical evidence suggests that polyphenol-rich dietary patterns are associated with a lower risk of cognitive decline, although findings are not fully consistent and causality cannot be established [27]. In particular, the Mediterranean and MIND diets—characterized by high intake of polyphenol-rich foods such as berries, leafy vegetables, olive oil, and nuts—have been consistently associated with better cognitive performance and reduced dementia risk in longitudinal studies [28].

The MIND (Mediterranean-DASH Intervention for Neurodegenerative Delay) diet is a hybrid dietary pattern that combines elements of the Mediterranean and DASH diets, specifically designed to promote brain health. It emphasizes the consumption of green leafy vegetables, berries, nuts, whole grains, olive oil, fish, and legumes, while limiting red meat, butter, cheese, sweets, and fried or fast foods. Adherence to the MIND diet has been associated with slower cognitive decline and a reduced risk of Alzheimer’s disease in observational studies, although causality cannot be established [29,30].

Mechanistic studies, primarily derived from in vitro and animal models, suggest that polyphenols may modulate key hallmarks of aging. They may reduce oxidative stress through activation of endogenous antioxidant systems, including the nuclear factor erythroid 2–related factor 2 (Nrf2) signaling pathway [31], and attenuate inflammation via inhibition of nuclear factor kappa B (NF-κB) signaling [32]. In addition, several polyphenols (e.g., resveratrol, curcumin, and epigallocatechin gallate (EGCG)) have been reported in preclinical models to modulate nutrient-sensing pathways such as AMP-activated protein kinase (AMPK), the mechanistic target of rapamycin (mTOR), and sirtuin 1 (SIRT1) signaling [33], which regulate autophagy, mitochondrial function, and cellular senescence [34].

From a neurological perspective, certain polyphenols have been shown to cross the BBB in experimental models, although evidence in humans remains limited, and may influence neuronal signaling, synaptic plasticity, and neurogenesis [35]. Moreover, their biological effects may also be mediated through the gut–brain axis, as microbial metabolism generates bioactive compounds with systemic and neuroprotective effects [36,37].

The aim of this narrative review is to provide an integrated overview of the role of dietary polyphenols in modulating the molecular mechanisms of brain aging within the geroscience framework. The review summarizes their dietary sources, bioavailability, and metabolism, and examines key signaling pathways—including Nrf2, NF-κB, AMPK, mTOR, and SIRT1—through which polyphenols may exert neuroprotective effects. Furthermore, it discusses the role of the gut–brain axis and synthesizes evidence from epidemiological and clinical studies linking dietary polyphenols to cognitive health.

## 2. Methods

A structured literature search was conducted in PubMed, Scopus, and Web of Science to identify relevant studies on dietary polyphenols, brain aging, and neurodegeneration up to December 2025. Search terms included combinations of keywords such as “polyphenols”, “brain aging”, “neurodegeneration”, “cognitive decline”, “gut–brain axis”, and “microbiome”, using Boolean operators (AND/OR).

Inclusion criteria prioritized peer-reviewed studies published in English, focusing on mechanistic, epidemiological, and clinical outcomes relevant to brain aging and neurodegeneration. Priority was given to high-quality evidence, including systematic reviews, meta-analyses, randomized controlled trials, and large prospective cohort studies. Mechanistic studies (in vitro and in vivo) were included to provide biological context and support interpretation of clinical findings.

Studies were selected based on relevance, methodological quality, and recency. Particular emphasis was placed on studies addressing molecular mechanisms, bioavailability, and clinically relevant cognitive outcomes. Reference lists of key articles were also screened to identify additional relevant publications. Although this is a narrative review, elements of a structured approach were applied to enhance transparency and reproducibility; however, no formal systematic review protocol was followed.

Data sharing is not applicable to this article, as no new data were created or analyzed in this study. The authors used ChatGPT (version 5.2; OpenAI, San Francisco, CA, USA) solely for language editing and improving the clarity of the manuscript. The tool was not used for data analysis, data interpretation, or the generation of scientific content. All content was critically reviewed and approved by the authors, who take full responsibility for the final manuscript.

## 3. Geroscience Framework: Biological Hallmarks of Brain Aging

Geroscience is an interdisciplinary field that examines the fundamental biological mechanisms of aging and their contribution to age-related chronic diseases [38]. A central premise of this framework is that seemingly distinct conditions—including cardiovascular, metabolic, and neurodegenerative diseases—share common cellular and molecular mechanisms driven by biological aging [39]. These processes are collectively referred to as the “hallmarks of aging” [5].

In the context of brain aging, key mechanisms include oxidative stress, chronic low-grade inflammation (inflammaging), mitochondrial dysfunction, cellular senescence, impaired proteostasis, and dysregulation of nutrient-sensing pathways. These interconnected processes act synergistically, driving progressive neuronal dysfunction and neurodegeneration.

**Figure 1:**
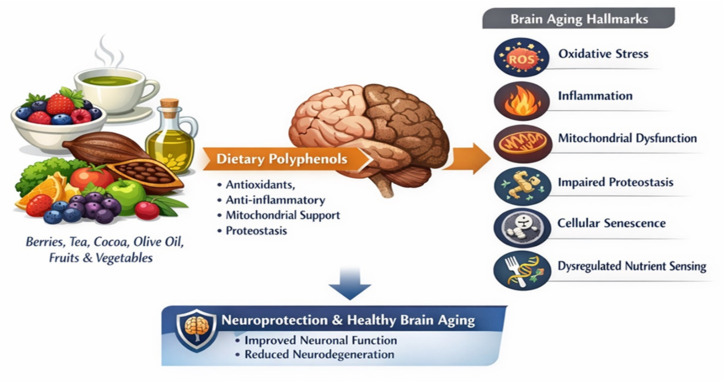
Proposed mechanisms by which dietary polyphenols may influence the hallmarks of brain aging, based primarily on preclinical evidence. The arrow represents proposed associations and mechanistic pathways linking dietary polyphenols to key hallmarks of brain aging and potential neuroprotective outcomes.

Emerging evidence, largely derived from preclinical studies, suggests that dietary bioactive compounds—particularly polyphenols—may modulate multiple hallmarks of brain aging through coordinated effects on cellular signaling networks. The interactions between dietary polyphenols and the hallmarks of brain aging, based primarily on experimental and preclinical evidence, are summarized in Figure 1.

This conceptual framework illustrates how polyphenols interact with multiple hallmarks simultaneously.

Aging of the nervous system is a complex, multifactorial process involving multiple interacting mechanisms [40,41,42]. Due to their high energy demand, long lifespan, and limited regenerative capacity, neurons are particularly vulnerable to cumulative molecular damage during aging [43,44]. These interconnected hallmarks include processes such as oxidative stress and inflammaging, which are discussed in detail below, collectively driving progressive neuronal dysfunction and neurodegeneration [45,46,47,48,49,50,51].

### 3.1. Oxidative Stress and Redox Imbalance

Oxidative stress is a fundamental mechanism of aging and occurs when the production of reactive oxygen species (ROS) and reactive nitrogen species (RNS) exceeds the capacity of endogenous antioxidant defenses. ROS are primarily generated during mitochondrial oxidative phosphorylation, although additional sources include enzymatic systems such as nicotinamide adenine dinucleotide phosphate (NADPH) oxidases [52,53].

The brain is particularly susceptible to oxidative damage due to its high oxygen consumption, the abundance of polyunsaturated fatty acids prone to lipid peroxidation, and a relatively limited antioxidant capacity, especially within glutathione-based systems [53,54,55,56]. Excess ROS can damage lipids, proteins, and nucleic acids, thereby impairing cellular function. Oxidative DNA lesions, such as 8-oxo-deoxyguanosine, contribute to genomic instability [57]. In addition, oxidative stress promotes pathological protein aggregation, including amyloid-β deposition in Alzheimer’s disease and α-synuclein accumulation in Parkinson’s disease [58].

### 3.2. Chronic Inflammation and Inflammaging

Aging is associated with a chronic, low-grade inflammatory state termed inflammaging [59], which is closely linked to age-related alterations in immune function (immunosenescence) [60]. This condition is characterized by persistently elevated circulating levels of pro-inflammatory mediators, including interleukin-6 (IL-6), tumor necrosis factor-α (TNF-α), and C-reactive protein (CRP) [61].

Within the central nervous system (CNS), microglia act as the primary mediators of immune responses and play a key role in maintaining neuronal homeostasis [62,63]. With aging, microglial activation becomes dysregulated, resulting in a lower activation threshold and exaggerated inflammatory responses [64]. Chronically activated microglia release cytokines, chemokines, and reactive oxygen species, thereby contributing to neuronal damage and neurotoxicity [64].

Inflammaging is also associated with impaired blood–brain barrier (BBB) integrity, facilitating the entry of peripheral inflammatory mediators into the CNS [10]. This further amplifies neuroinflammation and accelerates neurodegenerative processes [65].

### 3.3. Mitochondrial Dysfunction

Mitochondria are central to cellular energy metabolism through the generation of adenosine triphosphate (ATP) via oxidative phosphorylation [66]. With aging, mitochondrial function progressively declines, resulting in reduced ATP production and increased reactive oxygen species (ROS) generation [5,67,68,69]. A key contributor to this process is the accumulation of damage and mutations in mitochondrial DNA (mtDNA) [70].

Due to its proximity to the electron transport chain and its limited protective mechanisms compared with nuclear DNA, mtDNA is particularly susceptible to oxidative damage [71]. This further impairs electron transport chain function, creating a self-perpetuating cycle of ROS production and mitochondrial dysfunction [72,73,74].

Given the high energy demands of neurons, mitochondrial dysfunction has profound effects on neuronal physiology [75,76]. ATP depletion impairs synaptic transmission and may activate apoptotic pathways. For instance, the opening of mitochondrial permeability transition pores can trigger cytochrome c release and subsequent activation of the caspase cascade [77,78].

### 3.4. Cellular Senescence

Cellular senescence is defined as an irreversible cell-cycle arrest induced by stressors such as DNA damage, oxidative stress, and telomere shortening [79]. Although senescent cells remain metabolically active, they lose their proliferative capacity and accumulate progressively in aging tissues [80].

A hallmark feature of senescent cells is the senescence-associated secretory phenotype (SASP), characterized by the release of pro-inflammatory cytokines, growth factors, proteases, and other signaling molecules [81,82]. In the central nervous system, senescent astrocytes and microglia contribute to persistent neuroinflammation and tissue dysfunction. SASP factors can disrupt neuronal networks and promote the progression of neurodegenerative processes [83].

### 3.5. Impaired Proteostasis

Protein homeostasis (proteostasis) refers to the cellular capacity to maintain proper protein synthesis, folding, trafficking, and degradation [68,84,85]. With aging, this system progressively deteriorates, leading to the accumulation of misfolded and aggregated proteins [86]. Proteostasis is maintained by coordinated systems, including molecular chaperones, the ubiquitin–proteasome system, and the autophagy–lysosome pathway [87], which collectively preserve proteome integrity.

A defining pathological feature of many neurodegenerative diseases is the accumulation of abnormal protein aggregates. In Alzheimer’s disease, extracellular amyloid-β plaques and intracellular neurofibrillary tangles composed of hyperphosphorylated tau are observed [88]. In Parkinson’s disease, intracellular aggregates of α-synuclein (Lewy bodies) are characteristic [89]. These proteinopathies reflect impaired proteostasis and represent central drivers of neuronal dysfunction and degeneration.

### 3.6. Dysregulation of Nutrient-Sensing Signaling Pathways

Aging is also characterized by dysregulation of nutrient-sensing pathways that govern cellular energy balance, metabolism, and stress responses [90]. The mechanistic target of rapamycin (mTOR) signaling pathway plays a central role in regulating cell growth, protein synthesis, and autophagy [91]. Chronic activation of mTOR suppresses autophagy, thereby promoting the accumulation of damaged proteins and accelerating aging-related processes [92].

In contrast, AMP-activated protein kinase (AMPK) functions as a cellular energy sensor activated under conditions of energy deficiency. AMPK promotes catabolic pathways that restore ATP levels and metabolic homeostasis [93]. Its activation has been associated with extended lifespan and improved metabolic function primarily in experimental models [94].

Sirtuins, particularly sirtuin-1 (SIRT1), are nicotinamide adenine dinucleotide (NAD^+^)-dependent deacetylases involved in mitochondrial biogenesis, DNA repair, and cellular stress responses [95,96]. Disruption of the balance among these interconnected signaling pathways contributes to metabolic dysregulation, cellular dysfunction, and the development of neurodegenerative diseases [97,98]. Together, these hallmarks represent interconnected biological targets that may be influenced by dietary factors, although direct evidence in humans remains limited.

## 4. Dietary Polyphenols as Food-Derived Bioactive Compounds

Plant-derived foods contain a wide range of bioactive compounds that extend beyond the classical roles of macronutrients and micronutrients. These compounds, commonly referred to as phytochemicals, include polyphenols and other secondary metabolites with diverse biological activities. Increasing evidence from epidemiological and experimental studies suggests that regular consumption of plant-based foods is associated with a reduced risk of chronic diseases, including cardiovascular, metabolic, and neurodegenerative disorders [99].

Polyphenols represent one of the largest and most structurally diverse classes of these compounds. They are characterized by aromatic rings with one or more hydroxyl groups and play essential roles in plant defense against oxidative stress, ultraviolet radiation, and pathogens [100]. In the human diet, polyphenols are primarily derived from plant-based foods [101]. More than 5000 polyphenolic compounds have been identified, although many remain incompletely characterized [102]. Interest in polyphenols has increased substantially due to their potential role in the prevention and modulation of age-related diseases, including neurodegeneration [103]. Their biological effects are mediated through multiple mechanisms, including antioxidant activity, modulation of intracellular signaling pathways, and epigenetic regulation of gene expression.

### 4.1. Classification of Polyphenols

Polyphenols are classified into four major groups based on their chemical structure: flavonoids, phenolic acids, stilbenes, and lignans [104]. These structural differences influence their bioavailability, metabolism, and biological activity.

#### 4.1.1. Flavonoids

Flavonoids are the largest and most extensively studied subclass of polyphenols, characterized by a C6–C3–C6 structure consisting of two aromatic rings linked by a heterocyclic ring [105]. They include several subclasses, such as flavonols, flavones, flavanones, flavan-3-ols, isoflavones, and anthocyanins [106].

Representative compounds include quercetin and anthocyanins (e.g., cyanidin, delphinidin), which are commonly present in plant-based foods, particularly berries [107]. Importantly, the biological activity of flavonoids is strongly influenced by their bioavailability, which depends on factors such as glycosylation, intestinal absorption, and microbial metabolism. As a result, circulating metabolites often differ substantially from the parent compounds. Beyond their antioxidant and anti-inflammatory properties, flavonoids may exert neuroprotective effects through modulation of neuronal signaling pathways [108].

#### 4.1.2. Phenolic Acids

Phenolic acids primarily include hydroxybenzoic and hydroxycinnamic acid derivatives, such as caffeic, ferulic, and p-coumaric acids [109]. These compounds are widely distributed in plant-derived foods [110]. In addition to antioxidant activity, phenolic acids contribute to the regulation of redox homeostasis and modulation of inflammatory processes [111].

#### 4.1.3. Stilbenes

Stilbenes are a smaller but biologically important class of polyphenols, with resveratrol as the most prominent example. It is primarily found in grapes and related plant sources and has been widely studied for its potential geroprotective and neuroprotective effects [112]. Mechanistically, resveratrol has been shown to modulate aging-related pathways, including activation of sirtuin-1 (SIRT1), as well as mitochondrial function and oxidative stress responses [113].

#### 4.1.4. Lignans

Lignans are polyphenolic compounds predominantly found in plant-based foods, particularly seeds and whole grains [114]. They can be metabolized by the gut microbiota into enterolignans, which exhibit hormone-like and antioxidant effects [115]. These metabolites may interact with cellular signaling pathways and contribute to the modulation of age-related disease risk [115].

### 4.2. Major Dietary Sources of Polyphenols

Polyphenols are widely distributed across plant-derived foods, although their content varies depending on species, cultivation conditions, and processing methods [116,117]. Key sources include berries (anthocyanins), tea (catechins), cocoa (flavan-3-ols), olive oil (hydroxytyrosol), and coffee (chlorogenic acids). Importantly, the physiological effects of these compounds depend not only on their dietary presence but also on their bioavailability, metabolic transformation, and interaction with the gut microbiota. Consequently, exposure at the systemic level may differ substantially from dietary intake, which complicates interpretation of their biological effects.

Table 1 provides an overview of major dietary sources together with bioavailability and evidence levels, whereas Table 2 summarizes selected compounds and their molecular targets and signaling pathways involved in brain aging. Detailed evidence from epidemiological and clinical studies is presented in Section 8.

### 4.3. Polyphenol-Rich Dietary Patterns

Polyphenol intake is best understood within the context of dietary patterns rather than isolated compounds. The Mediterranean diet and the MIND diet (Mediterranean–DASH Intervention for Neurodegenerative Delay) are among the most extensively studied models in this regard [118].

The Mediterranean diet is characterized by high consumption of plant-based foods, including vegetables, fruits, legumes, nuts, and whole grains, with extra-virgin olive oil as the primary fat source [14,119,120,121]. This pattern provides a high intake of polyphenols and has been consistently associated with reduced risk of cognitive decline and dementia [14,122].

The MIND diet, a hybrid of the Mediterranean and DASH dietary patterns, specifically targets brain health by emphasizing polyphenol-rich foods such as berries, leafy green vegetables, nuts, and olive oil [28,123]. These dietary patterns provide a complex mixture of bioactive compounds that may synergistically modulate aging-related mechanisms, including oxidative stress, inflammation, and metabolic regulation [13,121,124,125,126].

## 5. Bioavailability and Metabolism of Polyphenols

The biological effects of dietary polyphenols are critically dependent on their bioavailability and metabolic transformation. Although many polyphenols exhibit strong activity in vitro, their in vivo efficacy is determined by absorption, biotransformation, systemic distribution, and excretion [127]. Most dietary polyphenols occur in conjugated forms (e.g., glycosides or esters), which substantially influence their intestinal absorption and metabolic fate [128].

Following ingestion, polyphenols undergo extensive biotransformation throughout the gastrointestinal tract and in peripheral tissues. After absorption, metabolites are further processed in the liver and distributed systemically or excreted via urine and bile. Overall, polyphenol bioavailability is generally low and highly variable, influenced by molecular structure, food matrix, gut microbiota composition, and host genetic factors [127].

### 5.1. Gastrointestinal Absorption

Polyphenol absorption occurs primarily in the gastrointestinal tract. Most compounds are present as glycosides, requiring enzymatic hydrolysis prior to absorption. Intestinal enzymes, such as lactase phlorizin hydrolase, cleave glycosidic bonds to release aglycones, which are more lipophilic and can be absorbed via passive diffusion or transporter-mediated mechanisms [129].

A substantial fraction of polyphenols escapes absorption in the small intestine and reaches the colon, where they are extensively metabolized by the gut microbiota [130]. These microbial transformations generate low-molecular-weight phenolic metabolites that are more readily absorbed and may significantly contribute to systemic biological effects. Absorbed compounds enter the portal circulation and undergo further hepatic metabolism [129].

### 5.2. Phase II Metabolism

Polyphenols are predominantly metabolized through phase II conjugation reactions in enterocytes and hepatocytes, resulting in more water-soluble metabolites that facilitate systemic circulation and excretion [131].

#### 5.2.1. Glucuronidation

Glucuronidation, catalyzed by UDP-glucuronosyltransferases, is a major metabolic pathway in which glucuronic acid is conjugated to hydroxyl groups, increasing solubility and promoting renal and biliary excretion. Glucuronide conjugates often represent the predominant circulating forms of polyphenols [128,132].

#### 5.2.2. Sulfation

Sulfation, mediated by sulfotransferases, further enhances solubility and contributes to systemic elimination. Sulfated metabolites are commonly detected in plasma and urine following dietary intake [133].

#### 5.2.3. Methylation

Methylation, catalyzed by catechol-O-methyltransferase, modifies hydroxyl groups of polyphenols, particularly catechol-containing compounds such as quercetin and caffeic acid. Although methylation may reduce direct antioxidant capacity, it can enhance metabolic stability and tissue distribution [134].

### 5.3. Role of the Gut Microbiota

The gut microbiota is a key determinant of polyphenol metabolism and bioactivity. Non-absorbed polyphenols reaching the colon undergo microbial transformations—including deglycosylation, dehydroxylation, demethylation, and aromatic ring cleavage—resulting in smaller phenolic metabolites such as phenylpropionic, phenylacetic, and benzoic acid derivatives [130,135]. These metabolites are often more bioavailable and may substantially contribute to systemic and neurobiological effects. Importantly, interindividual differences in microbiota composition lead to considerable variability in metabolic profiles and biological responses [136].

Polyphenols may also exert bidirectional effects by modulating the gut microbiota. Polyphenol-rich diets have been associated with increased abundance of beneficial bacterial genera, such as Lactobacillus and Bifidobacterium, suggesting prebiotic-like properties [137].

### 5.4. Transport Across the Blood–Brain Barrier

The neuroprotective potential of polyphenols depends in part on their ability to cross the blood–brain barrier (BBB), a highly selective interface regulating molecular entry into the central nervous system [138]. Certain low-molecular-weight polyphenols, particularly flavonoid aglycones, may cross the BBB via passive diffusion or transporter-mediated mechanisms, although evidence in humans remains limited [139]. Notably, circulating metabolites—rather than parent compounds—may reach higher concentrations in brain tissue [140,141]. Within the central nervous system, these compounds can modulate neuronal signaling pathways, attenuate oxidative stress and neuroinflammation, and support synaptic plasticity and neurogenesis [142].

### 5.5. Determinants of Bioavailability

Polyphenol bioavailability is shaped by multiple interacting factors, contributing to substantial interindividual variability [135,143].

#### 5.5.1. Food Matrix

The food matrix influences polyphenol release, stability, and absorption. Interactions with dietary fiber, proteins, and lipids may alter bioaccessibility, while co-ingestion with fats can enhance absorption of lipophilic compounds [143].

#### 5.5.2. Genetic Factors

Genetic polymorphisms affecting metabolic enzymes (e.g., UDP-glucuronosyltransferases, sulfotransferases, catechol-O-methyltransferase) contribute to interindividual variability in polyphenol metabolism and systemic exposure [144].

#### 5.5.3. Microbiome Composition

The gut microbiota plays a central role in determining polyphenol metabolism. Differences in microbial composition influence metabolite profiles and, consequently, biological activity [145]. Representative polyphenols, their dietary sources, molecular targets, and associated signaling pathways involved in neuroprotection are summarized in Table 2.

## 6. Molecular Mechanisms of Polyphenol-Mediated Neuroprotection

Accumulating evidence, largely derived from in vitro and animal studies, suggests that dietary polyphenols may exert neuroprotective effects through the coordinated modulation of interconnected molecular networks rather than isolated mechanisms. Beyond direct radical scavenging, these compounds have been shown in preclinical models to regulate redox homeostasis, neuroinflammatory signaling, mitochondrial function, proteostasis, and neurotrophic pathways, collectively contributing to neuronal resilience and functional integrity [146,147]. These processes are central to the pathophysiology of neurodegenerative disorders—including Alzheimer’s and Parkinson’s diseases—where oxidative stress, chronic inflammation, mitochondrial dysfunction, and protein aggregation converge to drive progressive neuronal loss [148,149].

**Figure 2:**
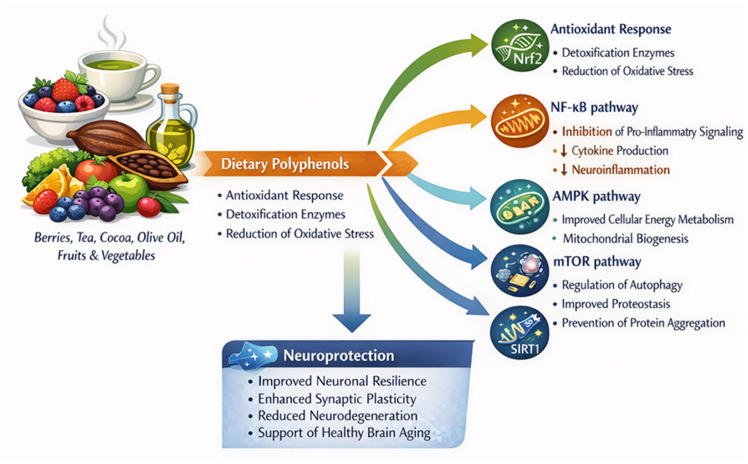
Molecular signaling pathways involved in polyphenol-mediated neuroprotection. Arrows represent proposed associations and mechanistic pathways through which dietary polyphenols may influence key signaling pathways, contributing to potential neuroprotective outcomes.

Importantly, the neurobiological activity of polyphenols is strongly shaped by their metabolic transformation and systemic distribution. Following ingestion, extensive phase II metabolism and gut microbiota-mediated biotransformation generate a diverse spectrum of circulating metabolites, some of which may access the central nervous system and influence neuronal signaling networks. As summarized in Figure 2, polyphenols have been shown to influence, primarily in experimental systems, key intracellular pathways involved in oxidative stress responses, inflammation, energy metabolism, proteostasis, and synaptic function.

The figure illustrates the convergence of redox, inflammatory, metabolic, and neurotrophic signaling pathways (Nrf2, NF-κB, AMPK, mTOR, SIRT1, BDNF) through which dietary polyphenols may exert pleiotropic neuroprotective effects, based predominantly on preclinical evidence.

### 6.1. Antioxidant Signaling Pathways

Oxidative stress is a key driver of neuronal damage in aging and neurodegeneration [150,151]. Although polyphenols can directly scavenge reactive oxygen species (ROS), their predominant antioxidant effects arise from activation of endogenous defense systems. A central regulator of this response is nuclear factor erythroid 2–related factor 2 (Nrf2) [152]. Under basal conditions, Nrf2 is sequestered in the cytoplasm by Kelch-like ECH-associated protein 1 (Keap1). Upon activation by oxidative stimuli or by polyphenols in experimental systems, Nrf2 has been shown to dissociate from Keap1 and translocate to the nucleus, where it binds antioxidant response elements (ARE) and induces transcription of cytoprotective genes [146,153,154]. These include enzymes such as superoxide dismutase, catalase, glutathione peroxidase, and heme oxygenase-1, collectively enhancing cellular redox capacity [155]. However, most evidence supporting Nrf2 activation by polyphenols derives from preclinical models, and clinical validation remains limited. Therefore, the translational relevance of these findings should be interpreted with caution.

### 6.2. Anti-Inflammatory Mechanisms

Chronic neuroinflammation is a hallmark of neurodegenerative disorders and is primarily mediated by activated microglia and astrocytes [156]. A key molecular target of polyphenols is nuclear factor kappa B (NF-κB), a master regulator of inflammatory gene expression [157]. Activation of NF-κB promotes transcription of pro-inflammatory mediators, including tumor necrosis factor-α, interleukin-1β, interleukin-6, inducible nitric oxide synthase, and cyclooxygenase-2 [158]. Polyphenols have been consistently reported in preclinical studies to inhibit NF-κB signaling, thereby potentially attenuating cytokine production and limiting sustained microglial activation [32]. In addition, polyphenols may modulate mitogen-activated protein kinase pathways, further contributing to the suppression of inflammatory signaling cascades. While these anti-inflammatory effects are consistently observed in experimental systems, human evidence remains heterogeneous and limited, highlighting the need for well-controlled clinical studies.

### 6.3. Mitochondrial Protection and Energy Metabolism

Mitochondrial dysfunction is a central feature of neuronal aging. Preclinical evidence suggests that polyphenols may support mitochondrial integrity through complementary mechanisms, including reduction of mitochondrial ROS production, improvement of respiratory efficiency, and stimulation of mitochondrial biogenesis [159].

A key regulator of mitochondrial biogenesis is peroxisome proliferator-activated receptor gamma coactivator 1-alpha (PGC-1α), which controls transcriptional programs involved in energy metabolism [160]. In parallel, polyphenols have been reported to activate AMP-activated protein kinase (AMPK) in experimental models, a critical cellular energy sensor that promotes metabolic adaptation, enhances mitochondrial function, and mitigates cellular stress [161]. Through coordinated modulation of PGC-1α and AMPK signaling, polyphenols may contribute to the maintenance of neuronal energy homeostasis. It should be noted that these effects have been predominantly demonstrated in vitro and in animal models, and their magnitude in humans remains to be established.

### 6.4. Regulation of Autophagy and Proteostasis

Efficient clearance of damaged proteins and organelles is essential for neuronal survival. Autophagy represents a key degradative pathway that maintains proteostasis [162]. The mechanistic target of rapamycin (mTOR) pathway is a major negative regulator of autophagy [163]. Hyperactivation of mTOR suppresses autophagic flux, facilitating the accumulation of misfolded proteins. Several polyphenols have been reported in experimental models to modulate mTOR signaling, thereby potentially promoting autophagy and enhancing the clearance of toxic protein aggregates. This mechanism is particularly relevant in neurodegenerative diseases characterized by amyloid-β and α-synuclein accumulation [163]. Although modulation of autophagy represents a promising mechanism, clinical evidence supporting this pathway in humans remains limited.

### 6.5. Synaptic Plasticity and Neurotrophic Signaling

Polyphenols may also influence neuronal survival and cognitive function by modulating neurotrophic pathways. Brain-derived neurotrophic factor (BDNF) is a central mediator of synaptic plasticity, learning, and memory [164]. Activation of BDNF signaling through its receptor TrkB triggers downstream cascades, including PI3K/Akt, MAPK/ERK, and phospholipase C pathways, ultimately leading to activation of CREB [165]. Polyphenols, particularly flavonoids, have been reported in experimental models to enhance BDNF expression and CREB phosphorylation, thereby potentially supporting synaptic function and neuronal resilience during aging [164].

### 6.6. Epigenetic Regulation

Emerging evidence from experimental studies indicates that polyphenols may modulate gene expression through epigenetic mechanisms, including histone modifications and DNA methylation [141].

Polyphenols may influence histone acetylation by regulating histone acetyltransferases and histone deacetylases, thereby altering chromatin accessibility and transcriptional activity [166,167]. In addition, modulation of DNA methyltransferase activity may affect DNA methylation patterns associated with oxidative stress, inflammation, and neuronal survival pathways [168,169]. These epigenetic effects represent a plausible mechanism for sustained modulation of cellular processes, linking dietary exposures to longer-term changes in gene expression and neuronal function. Overall, while these molecular mechanisms provide strong biological plausibility, the majority of supporting evidence is derived from preclinical models, and their relevance at physiologically achievable exposure levels in humans remains to be clearly established.

## 7. Polyphenols and Neurodegenerative Diseases

Accumulating evidence from experimental, epidemiological, and limited clinical studies suggests that dietary polyphenols may modulate key molecular processes underlying neurodegeneration. Rather than acting through a single mechanism, these compounds appear to exert pleiotropic effects—primarily demonstrated in non-human systems—including attenuation of oxidative stress, suppression of neuroinflammation, inhibition of pathological protein aggregation, and modulation of signaling pathways governing neuronal survival and synaptic plasticity [147,170,171,172]. Available evidence further indicates that specific polyphenols, such as resveratrol, curcumin, quercetin, and epigallocatechin gallate (EGCG), may target core pathogenic pathways shared across neurodegenerative disorders [11,12,18,103].

### 7.1. Alzheimer’s Disease

Alzheimer’s disease is the most prevalent form of dementia and is characterized by progressive cognitive decline and synaptic dysfunction [173]. Its neuropathology is defined by extracellular amyloid-β (Aβ) deposition, intracellular neurofibrillary tangles composed of hyperphosphorylated tau, and sustained neuroinflammation [174]. Polyphenols have been reported to interact with multiple components of this pathogenic cascade, predominantly based on preclinical evidence, whereas human data remain limited and heterogeneous.

#### 7.1.1. Amyloid-β Aggregation

Aβ peptides, generated from amyloid precursor protein (APP), aggregate into oligomers and fibrils that disrupt synaptic function and induce neurotoxicity [175]. Several polyphenols have been shown in experimental models to interfere with Aβ aggregation by modulating both nucleation and fibril elongation [176]. Compounds such as curcumin, myricetin, and gallic acid have been reported to stabilize less toxic conformations of Aβ and reduce oligomer formation [177]. In addition, certain flavonoids may directly interact with Aβ, altering its structural dynamics and inhibiting fibrillization [177]. Polyphenols may also enhance Aβ clearance through activation of autophagic and proteasomal pathways [178], although these mechanisms have been primarily characterized in experimental systems.

#### 7.1.2. Tau Hyperphosphorylation

Tau pathology represents a second major driver of AD progression. Hyperphosphorylation of tau leads to microtubule destabilization and formation of neurofibrillary tangles [179]. Polyphenols such as resveratrol and curcumin have been shown in experimental studies to modulate tau-related signaling by inhibiting glycogen synthase kinase-3β (GSK-3β) and activating sirtuin 1 (SIRT1) pathways [180]. These effects may contribute to preserving cytoskeletal integrity and potentially mitigating neurodegenerative progression, although supporting evidence is largely preclinical [181].

#### 7.1.3. Neuroinflammation

Chronic activation of microglia and astrocytes amplifies neurodegeneration in AD [182]. Polyphenols may attenuate neuroinflammatory responses primarily through inhibition of nuclear factor kappa B (NF-κB) signaling, thereby potentially reducing the expression of pro-inflammatory cytokines such as tumor necrosis factor-α and interleukin-1β [32]. Additionally, compounds such as EGCG have been reported in experimental models to promote a shift in microglial phenotype toward a more anti-inflammatory state [183], although clinical confirmation remains limited.

### 7.2. Parkinson’s Disease

Parkinson’s disease is characterized by progressive loss of dopaminergic neurons in the substantia nigra and impaired dopaminergic signaling within the basal ganglia [184]. Clinically, PD manifests with motor symptoms including tremor, rigidity, and bradykinesia [185]. Polyphenols may influence key pathogenic mechanisms involved in PD progression, although current evidence is derived predominantly from experimental studies.

#### 7.2.1. α-Synuclein Aggregation

Aggregation of α-synuclein into Lewy bodies represents a central pathological feature of PD [186]. Polyphenols have been shown in experimental models to modulate α-synuclein aggregation dynamics by stabilizing monomeric forms and preventing formation of toxic oligomers [187]. For example, quercetin and resveratrol have been reported to reduce aggregation and associated neurotoxicity, partly through antioxidant and protein-interacting properties [188], although these findings are largely limited to in vitro and animal studies.

#### 7.2.2. Degeneration of Dopaminergic Neurons

Degeneration of dopaminergic neurons is driven by oxidative stress, mitochondrial dysfunction, and inflammation [189]. Polyphenols may counteract these processes by reducing reactive oxygen species (ROS) production, preserving mitochondrial function, and modulating apoptotic signaling pathways, as demonstrated primarily in experimental models [190]. In addition, epidemiological studies suggest that higher intake of flavonoid-rich foods, particularly anthocyanin-rich berries, is associated with a lower risk of PD [191,192], although causal relationships cannot be established.

### 7.3. Cognitive Aging and Mild Cognitive Impairment

Mild cognitive impairment (MCI) represents an intermediate stage between normal aging and dementia, characterized by measurable cognitive decline with preserved functional independence [193]. Emerging evidence from observational and interventional studies suggests that polyphenol-rich diets may support cognitive function and may be associated with delayed progression toward dementia.

Flavonoid-rich foods—including berries, cocoa, and green tea—have been associated with improvements in memory, attention, and executive function, although effect sizes are generally modest and findings are not fully consistent [194,195]. Mechanistically, these effects may involve enhanced cerebral blood flow, improved endothelial function, and activation of neurotrophic signaling pathways, with mechanistic insights largely derived from experimental research [35]. In particular, polyphenols have been shown to upregulate brain-derived neurotrophic factor (BDNF), a key regulator of synaptic plasticity and neuronal survival, predominantly in non-human models [164].

Clinical studies further indicate that polyphenol-rich interventions may be associated with improvements in episodic memory and executive performance in older adults, especially in individuals with early cognitive decline [196], although heterogeneity across studies limits definitive conclusions. Collectively, these findings suggest a potential role for dietary polyphenols in supporting cognitive function; however, well-designed, adequately powered clinical trials are required to confirm these effects.

## 8. Evidence from Epidemiological and Clinical Studies

### 8.1. Prospective Cohort Studies

Prospective cohort studies provide moderate but generally consistent evidence on the long-term health effects of flavonoid and polyphenol intake. Data from large European cohorts indicate that individual flavonoid subclasses may differentially influence chronic disease risk. Within the EPIC studies, higher intakes of specific flavanols and flavonols were inversely associated with type 2 diabetes incidence [197], whereas no consistent association was observed between flavan-3-ol intake and cardiovascular risk [198]. Biomarker-based analyses further support a protective role, as higher urinary polyphenol concentrations were associated with reduced all-cause mortality in older adults [199].

With respect to cognitive outcomes, higher adherence to flavonoid-rich dietary patterns has been associated with lower dementia risk in large population-based cohorts [200]. However, findings across longitudinal studies remain heterogeneous, with some reporting only modest or non-significant associations with cognitive decline [201]. Notably, dietary patterns enriched in lignans, flavonols, and isoflavonoids have been linked to more favorable cognitive trajectories and reduced dementia risk [202,203]. Additional evidence suggests that higher intake of flavonoid-rich fruits—particularly during midlife—and long-term consumption of specific subclasses such as flavonols and anthocyanins are associated with lower risk of dementia and Alzheimer’s disease [204,205]. Overall, these findings suggest that flavonoid intake—especially when considered at the level of subclasses and dietary patterns—may beneficially influence cardiometabolic and cognitive outcomes, although residual confounding and heterogeneity limit causal inference.

### 8.2. Dietary Pattern Studies

Evidence from dietary pattern analyses suggests that the cognitive effects of polyphenols are best understood within the context of whole diets rather than isolated compounds. Observational studies and meta-analyses indicate that higher flavonoid intake is generally associated with better cognitive performance and reduced risk of cognitive decline, albeit with variability across studies [27]. Mechanistically, these associations may reflect combined effects on oxidative stress, inflammation, cerebral perfusion, and neuronal plasticity. However, interindividual variability in bioavailability and blood–brain barrier permeability likely contributes to inconsistencies [103].

At the population level, insufficient fruit and vegetable intake remains a major public health concern and may contribute to increased risk of chronic diseases and cognitive decline [206]. Higher adherence to the Mediterranean diet has been consistently associated with lower risk of cognitive disorders, with evidence of a dose–response relationship [207]. Similarly, emerging data on the MIND diet support its role in slowing cognitive decline and delaying neurodegenerative processes [208]. Collectively, these findings support a synergistic model in which multiple bioactive compounds within dietary patterns interact to promote cognitive health.

### 8.3. Clinical Intervention Trials

Randomized controlled trials provide emerging, yet still limited, evidence that polyphenol-rich interventions may influence specific domains of cognitive function, although effect sizes are generally modest. Anthocyanin-rich berry interventions have demonstrated improvements in memory and executive function, as well as maintenance of cognitive performance under acute cognitive load [209,210,211]. While not all outcomes reach statistical significance, benefits are most consistently observed in working memory domains [209].

Cocoa flavanol interventions have yielded some of the most consistent findings, with improvements reported in executive function, processing speed, verbal fluency, and hippocampal-dependent memory [212,213,214,215]. These effects may be partly mediated by improvements in vascular and metabolic parameters, including blood pressure and insulin sensitivity. However, null findings in some acute studies highlight dose- and population-dependent variability [216].

Other compounds also show potential. Soy isoflavones have been associated with modest improvements in global cognition and visual memory [217], while plant-derived extracts such as green oat have demonstrated acute benefits in attention and executive function [218].

More recent trials suggest that polyphenol-rich nutraceuticals may be associated with enhanced cognitive performance and modulation of neuroplasticity-related biomarkers, including brain-derived neurotrophic factor (BDNF) and cAMP response element-binding protein (CREB) [219]. Meta-analyses indicate small-to-moderate but generally positive effects, particularly when adequate dosing and bioavailability are achieved [220]. Notably, anthocyanin-rich interventions have been associated with improvements across multiple cognitive domains [221]. Overall, clinical evidence supports a potentially beneficial role of polyphenols in cognitive health; however, heterogeneity in study design, dosage, and study populations remains a major limitation.

### 8.4. Cognitive Outcomes

Meta-analyses and systematic reviews generally indicate that flavonoids and polyphenols are associated with modest but statistically significant improvements in cognitive function, particularly in memory, processing speed, and mood-related domains. A large meta-analysis of randomized controlled trials reported modest improvements in global cognitive performance, with the strongest effects observed for cocoa, berries, and selected plant extracts [222].

These associations may be partly explained by converging biological mechanisms, including attenuation of oxidative stress and neuroinflammation, modulation of neuronal signaling pathways, and enhancement of neuroplasticity—particularly via upregulation of BDNF [108]. In addition, polyphenols have been suggested to inhibit protein aggregation and improve cerebrovascular function, both of which are highly relevant to neurodegenerative processes [108,223].

Evidence further suggests that polyphenol supplementation may preferentially benefit specific cognitive domains, such as immediate memory, particularly in at-risk populations (e.g., older adults or individuals with metabolic dysfunction), although responses are not uniform across domains [224]. Emerging approaches—including polyphenol-rich functional foods, nutraceuticals, and concepts such as nutritional senotherapeutics—highlight the potential of targeting aging-related pathways to preserve cognitive function [225,226].

Overall, while epidemiological and clinical evidence suggests potentially beneficial associations between polyphenol intake and cognitive outcomes, substantial heterogeneity in study design, exposure assessment, and outcome definitions limits comparability and causal inference. Residual confounding, measurement error in dietary assessment, and interindividual variability further complicate interpretation. Therefore, current evidence should be interpreted with caution, and well-designed, adequately powered randomized controlled trials with standardized exposure assessment are required to establish causal relationships and clinical relevance.

Importantly, while the current body of evidence predominantly supports a preventive role of polyphenol-rich dietary patterns, emerging data suggest that these interventions may also exert adjunctive therapeutic effects, particularly in early or prodromal stages of neurodegenerative disorders; however, clinical evidence remains limited and heterogeneous [21]. Moreover, cognitive aging and neurodegeneration are multifactorial processes influenced by a complex interplay of lifestyle and biological factors, including physical activity, sleep quality, vascular health, metabolic status, and genetic predisposition [21]. Consequently, the effects of dietary polyphenols should be interpreted within the context of broader lifestyle patterns rather than as isolated interventions. Future strategies should therefore prioritize multidomain approaches integrating dietary modification with other evidence-based interventions—such as physical activity, cognitive training, and vascular risk management—which may provide synergistic benefits and represent a more effective strategy for preventing or delaying cognitive decline [21].

## 9. Gut–Brain Axis and Microbiome Interactions

The gut–brain axis represents a bidirectional communication network linking the gastrointestinal system, gut microbiota, immune system, and central nervous system through neural, endocrine, metabolic, and immunological pathways [227]. Increasing evidence suggests that dietary polyphenols may modulate this axis primarily via interactions with the gut microbiota, thereby influencing neurobiological processes relevant to brain aging and neurodegeneration [228].

The gut microbiota constitutes a metabolically active ecosystem capable of generating bioactive compounds that regulate host immunity, metabolism, and neuronal function [229]. The relationship between polyphenols and the microbiota is bidirectional: polyphenols shape microbial composition and activity, while microbial metabolism transforms polyphenols into bioactive metabolites. This polyphenol–microbiota–host interplay represents a key regulatory system influencing oxidative stress, inflammation, and energy metabolism [230].

### 9.1. Polyphenol–Microbiota Interactions

Only a small proportion of dietary polyphenols is absorbed in the small intestine, with the majority reaching the colon where they undergo extensive microbial biotransformation [231]. Microbial enzymes catalyze reactions such as hydrolysis, demethylation, and ring cleavage, generating metabolites with enhanced bioavailability and biological activity [232].

Conversely, polyphenols modulate microbiota composition by increasing microbial diversity and promoting beneficial taxa, including Bifidobacterium, Lactobacillus, and *Akkermansia muciniphila*, which contribute to gut barrier integrity, short-chain fatty acid production, and immune regulation [233]. Polyphenols may also suppress pathogenic bacteria, partly through antimicrobial effects and modulation of microbial signaling pathways [230].

### 9.2. Microbial Metabolites

Microbial metabolism of polyphenols generates bioactive metabolites such as phenolic acids, phenyl-γ-valerolactones, urolithins, dihydroresveratrol, and enterolignans [234]. These compounds are absorbed into the circulation and may reach the central nervous system, where they influence key signaling pathways, including nuclear factor erythroid 2–related factor 2 (Nrf2), nuclear factor kappa B (NF-κB), and AMP-activated protein kinase (AMPK) [235].

Through these mechanisms, microbial metabolites contribute to the regulation of oxidative stress, inflammation, and mitochondrial function. Notably, urolithins have been shown to activate mitophagy and improve mitochondrial health, highlighting their potential role in aging-related processes [236]. Representative metabolites and their biological effects are summarized in Table 3.

### 9.3. Modulation of Neuroinflammation

Microbiota-derived metabolites play a critical role in regulating neuroinflammation via the gut–brain axis. Polyphenol-derived metabolites can suppress inflammatory signaling by inhibiting NF-κB activation and reducing pro-inflammatory cytokine production, including tumor necrosis factor-α, interleukin-6, and interleukin-1β [237]. In parallel, activation of Nrf2-dependent pathways enhances antioxidant defenses [238]. Additionally, polyphenols contribute to the maintenance of intestinal barrier integrity, reducing translocation of lipopolysaccharides and systemic inflammation [239]. This mechanism is particularly relevant in neurodegenerative diseases, where peripheral inflammation may exacerbate central neuroinflammatory processes [240].

### 9.4. Microbiome-Mediated Neuroprotective Effects

Microbiome-derived metabolites, particularly short-chain fatty acids such as acetate, propionate, and butyrate, play a central role in neuroimmune regulation. These compounds modulate microglial activity, support blood–brain barrier integrity, and influence neurotrophic signaling [241,242].

Through these pathways, polyphenol-induced microbial changes may contribute to the enhancement of synaptic plasticity, neurogenesis, and mitochondrial function while reducing oxidative stress and inflammation [243]. Collectively, the polyphenol–microbiota axis represents a potentially important interface linking diet to brain health and a promising target for nutrition-based strategies aimed at preventing neurodegenerative diseases [244].

## 10. Challenges and Research Gaps

Despite substantial progress in elucidating the biological effects of dietary polyphenols, several methodological and conceptual limitations continue to hinder their translation into clinical and public health applications. These challenges primarily relate to bioavailability constraints, heterogeneity of exposure, limited high-quality clinical evidence, interindividual variability, and difficulties in accurately assessing intake.

### 10.1. Bioavailability and Dose–Response Considerations

A major challenge in polyphenol research is their low and highly variable bioavailability. Most dietary polyphenols are present as glycosides, esters, or polymeric forms, which require enzymatic or microbial transformation prior to absorption [116,245]. Consequently, circulating compounds predominantly consist of conjugated metabolites (e.g., glucuronides, sulfates, methylated derivatives) rather than native molecules.

Bioavailability is influenced by multiple factors, including molecular structure, food matrix interactions, intestinal transport mechanisms, gut microbiota composition, and metabolic enzyme activity [128]. As a result, only a small fraction of ingested polyphenols reaches systemic circulation in biologically relevant forms, complicating the interpretation of their biological effects.

Importantly, the characterization of dose–response relationships remains a major unresolved challenge in polyphenol research. Current evidence suggests that the relationship between intake and physiological response is often non-linear, context-dependent, and influenced by substantial interindividual variability. In many cases, biological effects observed in preclinical models occur at concentrations that are not achievable through habitual dietary intake.

A critical distinction should be made between different exposure levels:

- Habitual dietary intake, reflecting polyphenol consumption from whole foods within typical dietary patterns;
- Enhanced dietary intake, achievable through targeted dietary modification;
- Pharmacological or supplemental exposure, often involving concentrated extracts or purified compounds.

Moreover, circulating concentrations of native polyphenols are typically low, with biological effects largely attributable to their metabolites rather than parent compounds. This complicates dose standardization, as the link between intake, circulating metabolites, and biological activity remains incompletely defined [246]. A further limitation arises from the discrepancy between doses used in experimental models and those achievable in humans. Preclinical studies frequently apply supra-physiological concentrations, whereas human investigations are constrained to dietary or moderately elevated intake levels. This mismatch limits translational relevance and may contribute to inconsistent findings. Overall, the absence of clearly defined dose–response relationships, together with variability in bioavailability and metabolic transformation, remains an important barrier to establishing evidence-based dietary recommendations and clinical applications. These differences across exposure levels are summarized in Table 4. Notably, habitual dietary intake of individual polyphenols is substantially lower than doses used in intervention studies. For example, resveratrol intake from diet is typically below 5 mg/day, whereas supplementation studies employ doses in the range of 150–1000 mg/day [247,248,249].

### 10.2. Heterogeneity of Polyphenol Intake

Polyphenol intake varies widely across populations due to differences in dietary patterns, cultural habits, and food availability. In addition, the polyphenol content of foods is highly variable and influenced by plant variety, environmental conditions, and processing methods [257,258].

The increasing consumption of ultra-processed foods—typically low in polyphenols—may further reduce overall intake and contribute to inconsistencies in epidemiological findings [259]. This variability limits comparability across studies and complicates the establishment of standardized intake recommendations.

### 10.3. Lack of Large-Scale Clinical Trials

Although experimental and observational data support the neuroprotective potential of polyphenols, robust clinical evidence remains limited. Existing human intervention studies are often characterized by small sample sizes, short durations, and heterogeneous study designs [260,261].

Moreover, isolating the effects of individual polyphenols is challenging due to their consumption within complex dietary matrices [262]. Future research should prioritize adequately powered, long-term randomized controlled trials with standardized interventions, clearly defined doses, and clinically meaningful endpoints.

### 10.4. Interindividual Variability

Substantial interindividual variability in polyphenol metabolism and response represents a critical limitation. The gut microbiota plays a central role in generating bioactive metabolites, and its composition varies markedly between individuals [263].

Additional determinants—including genetic background, age, sex, metabolic status, and overall diet—further influence polyphenol bioactivity [264]. Consequently, individuals with similar intake levels may exhibit markedly different metabolic profiles and physiological responses, underscoring the need for personalized approaches in polyphenol research.

### 10.5. Limitations in Measuring Dietary Intake

Accurate assessment of polyphenol intake remains a major challenge in epidemiological studies. Most evidence relies on self-reported dietary data, which are prone to measurement error. Although polyphenol composition databases have improved, substantial variability in food content persists [265]. Biomarker-based approaches—such as the measurement of polyphenol metabolites in plasma or urine—offer a more objective assessment of exposure and are increasingly recognized as essential tools in nutritional epidemiology.

Importantly, future research should move beyond merely describing these limitations and increasingly focus on potential solutions. Strategies to improve clinical translation may include the development and validation of standardized polyphenol biomarkers, the integration of metabolomics-based exposure assessment, and the design of personalized interventions based on microbiome composition and genetic profiles. In addition, harmonization of study protocols and dose standardization across trials would improve comparability and strengthen causal inference.

Without accurate quantification of polyphenol intake, interpretation of individual-level effects remains highly uncertain, representing a major limitation in both epidemiological and clinical research. This variability may partly explain the inconsistent findings observed across clinical trials and underscores the need for precision nutrition approaches targeting specific responder subgroups. Furthermore, the lack of well-defined and standardized dose–response relationships remains a critical barrier to clinical translation and the development of evidence-based recommendations, particularly given the variability in bioavailability and metabolic response.

### 10.6. Timing and Duration of Polyphenol Intake

The timing and duration of dietary polyphenol intake represent important yet insufficiently explored determinants of their biological effects. Evidence indicates that plasma polyphenol levels are highly dynamic, typically peaking within 1–2 h after ingestion and declining thereafter, which may introduce substantial variability depending on fasting status and sampling conditions [255]. In contrast, long-term habitual intake appears to play a more relevant role in shaping sustained physiological responses. Long-term dietary interventions suggest that consistent polyphenol consumption, rather than acute intake, is associated with improvements in oxidative stress and inflammatory profiles, although responses may vary considerably between individuals [128].

This interindividual variability, often reflected in responder versus non-responder patterns, may be driven by differences in gut microbiota composition, metabolic capacity, genetic background, and baseline health status. Consequently, both the timing of intake and the duration of exposure should be considered when interpreting clinical and epidemiological findings. Future research should aim to better characterize these dynamics, including postprandial kinetics, long-term adherence, and personalized response patterns, in order to enhance the translational potential of polyphenol research.

### 10.7. Safety and Toxicity Considerations

Although dietary polyphenols are widely regarded as beneficial bioactive compounds, their safety profile warrants careful consideration, particularly in the context of high-dose supplementation and clinical translation. Current evidence indicates that most adverse effects of polyphenols have been observed in experimental settings, often at concentrations substantially exceeding those achievable through habitual dietary intake. At pharmacological doses, certain polyphenols may exert pro-oxidant activity, potentially leading to oxidative damage rather than protection. This paradoxical effect has been reported for compounds such as quercetin, particularly under conditions of high concentration or in the presence of transition metals [266,267].

Some polyphenols have also been associated with potential genotoxic or carcinogenic effects in preclinical models when administered at high doses [245]. In addition, specific subclasses, such as isoflavones, exhibit endocrine activity and may interfere with hormone regulation, including thyroid function and estrogen signaling. These effects appear to be dose-dependent and may be particularly relevant in vulnerable populations, such as infants, pregnant women, or individuals with micronutrient deficiencies [266].

Polyphenols may also exert antinutritional effects, most notably by inhibiting non-heme iron absorption, which could contribute to iron deficiency in populations with marginal iron status [268]. Furthermore, interactions with pharmaceutical agents represent an important but often underrecognized concern. Certain polyphenols can modulate drug-metabolizing enzymes (e.g., cytochrome P450), thereby altering drug bioavailability and pharmacokinetics [269].

Importantly, the safety profile of polyphenols differs substantially depending on their source and form. Intake from whole foods is generally considered safe, as exposure levels remain within physiological ranges and are modulated by the food matrix. In contrast, concentrated extracts and dietary supplements may result in substantially higher systemic exposure, potentially exceeding safe thresholds.

Overall, current evidence suggests that while dietary polyphenols are safe within the context of a balanced diet, caution is warranted when considering high-dose supplementation. Future research should prioritize dose-relevant safety assessments, particularly in human studies, and establish evidence-based upper intake levels to support safe clinical application.

## 11. Future Perspectives

Advances in nutritional science are increasingly enabling a systems-level understanding of how dietary bioactive compounds, particularly polyphenols, influence human health. Future research is expected to move beyond reductionist approaches toward integrative frameworks that combine genomics, metabolomics, and microbiome science to elucidate the complex, context-dependent effects of polyphenols. Such approaches are critical for translating mechanistic insights into targeted, effective, and scalable nutritional strategies for healthy aging and neuroprotection. To facilitate clinical translation, the major research gaps and priorities are summarized in Table 5. These priorities highlight key challenges that must be addressed to bridge the gap between mechanistic findings and human applications.

### 11.1. Precision Nutrition

Precision nutrition is founded on the premise that interindividual variability substantially modifies responses to dietary exposures. Genetic background, metabolic phenotype, and microbiome composition collectively determine the bioavailability, metabolism, and biological activity of polyphenols. Future strategies should integrate multi-dimensional individual data—including metabolic biomarkers, lifestyle factors, and clinical parameters—to optimize dietary recommendations. In the context of polyphenols, this approach may enable the identification of responders and non-responders, thereby improving intervention efficacy and advancing personalized prevention strategies for cardiometabolic and neurodegenerative diseases.

### 11.2. Nutrigenomics

Nutrigenomics provides critical insight into how polyphenols interact with gene regulatory networks. Polyphenols have been shown to modulate the expression of genes involved in oxidative stress responses, inflammation, and cellular energy metabolism, often through epigenetic and transcriptional mechanisms. Future research should focus on identifying gene–diet interactions and functional polymorphisms that influence polyphenol metabolism and signaling. Such knowledge may enable the development of genotype-informed nutritional interventions targeting key pathways such as AMP-activated protein kinase signaling, insulin sensitivity, and lipid metabolism.

### 11.3. Metabolomics-Based Approaches

Metabolomics offers a powerful platform for characterizing the dynamic metabolic responses to dietary polyphenols. By quantifying circulating and excreted metabolites, this approach enables objective assessment of exposure and biological activity. Importantly, many of the bioactive compounds detected in vivo are microbiota-derived metabolites rather than parent polyphenols. Integrating metabolomic profiling into clinical studies will improve the identification of functional biomarkers, facilitate dose–response evaluation, and enhance the prediction of individual responses to polyphenol-rich interventions.

### 11.4. Microbiome-Targeted Strategies

The gut microbiome is a central determinant of polyphenol metabolism and bioactivity. Future interventions are likely to target the microbiome to enhance the generation of beneficial metabolites and improve systemic effects. Potential strategies include prebiotic approaches to stimulate polyphenol-metabolizing bacteria, probiotic or postbiotic interventions to modulate microbial function, and dietary designs that optimize microbial–host interactions. Such approaches may substantially enhance the bioavailability and efficacy of polyphenols in neuroprotection.

### 11.5. Integrative Multi-Omics Approaches

The integration of genomics, transcriptomics, metabolomics, proteomics, and microbiome data represents a transformative direction in nutritional science. Multi-omics approaches enable the characterization of complex biological networks underlying diet–health interactions and provide a framework for identifying causal pathways.

In the context of polyphenols, such integrative analyses may uncover novel molecular targets, refine risk stratification, and support the development of precision dietary interventions aimed at delaying aging-related functional decline. Despite these advances, translation into clinically applicable strategies remains limited and requires validation in well-designed human studies.

## 12. Conclusions

Dietary polyphenols represent a biologically plausible and increasingly supported, yet not fully established, strategy for promoting healthy brain aging. Converging data from mechanistic, experimental, and human studies suggest that these compounds may modulate multiple interconnected pathways—including oxidative stress, neuroinflammation, mitochondrial dysfunction, impaired proteostasis, and neurotrophic signaling—thereby targeting core processes underlying neurodegeneration.

Epidemiological and clinical evidence further supports that polyphenol-rich dietary patterns are associated with improved cognitive outcomes and reduced risk of cognitive decline, although effect sizes remain modest and context-dependent. Importantly, the gut–brain axis has emerged as a key mechanistic interface linking dietary polyphenols, microbiota-derived metabolites, and central nervous system function.

Despite this growing body of evidence, several challenges continue to limit clinical translation. These include low and highly variable bioavailability, substantial interindividual variability driven by metabolic and microbiome differences, and the lack of adequately powered, long-term randomized controlled trials. Addressing these limitations will require standardized interventions, biomarker-based exposure assessment, and rigorously designed, mechanism-informed clinical studies.

Future progress will depend on the integration of precision nutrition, nutrigenomics, metabolomics, and microbiome research within a systems-level framework. Such approaches may enable personalized, mechanism-based dietary strategies that optimize the neuroprotective effects of polyphenols and contribute meaningfully to the prevention and delay of age-related cognitive decline.

## Figures and Tables

**Table 1:** Major dietary sources of polyphenols, bioavailability, and biological relevance to brain aging.

Polyphenol Class	Representative Compounds	Major Dietary Sources	Bioavailability	Evidence Level	Biological Relevance to Brain Aging
Flavonoids	Quercetin, catechin, epicatechin, anthocyanins	Berries, apples, onions, tea, cocoa	Low–moderate (structure-dependent)	Human and preclinical	Antioxidant and anti-inflammatory effects; modulation of neuronal signaling and synaptic plasticity
Phenolic acids	Caffeic acid, ferulic acid, chlorogenic acid	Coffee, fruits, vegetables, cereals	Moderate	Human and preclinical	Regulation of redox homeostasis; attenuation of oxidative stress and inflammation
Stilbenes	Resveratrol	Grapes, red wine, berries	Low (rapid metabolism)	Human and preclinical	Activation of SIRT1; mitochondrial function and metabolic regulation
Lignans	Secoisolariciresinol, matairesinol	Flaxseed, sesame seeds, whole grains	Low–moderate (microbiota-dependent)	Preclinical and limited human	Gut microbiota-derived metabolites; antioxidant and hormone-like effects

Abbreviations: SIRT1, sirtuin 1.

**Table 2:** Polyphenols, molecular targets, and translational relevance in brain aging.

Polyphenol	Major Dietary Sources	Molecular Target	Signaling Pathway	BBB Permeability	Evidence Level	Reported Neuroprotective Effects
Resveratrol	Grapes, red wine, berries	SIRT1 activation	SIRT1–AMPK axis	Yes (limited bioavailability)	Human + animal	Mitochondrial biogenesis, reduced oxidative stress, improved cognitive performance (small RCTs)
Epigallocatechin gallate (EGCG)	Green tea	Nrf2 activation	Nrf2–ARE pathway	Limited	Mostly preclinical	Enhanced antioxidant defense, reduced neuronal oxidative damage
Quercetin	Apples, onions, tea	NF-κB inhibition	NF-κB signaling	Low–moderate	Preclinical + limited human	Anti-inflammatory effects, attenuation of neuroinflammation
Curcumin	Turmeric	mTOR inhibition	mTOR–autophagy pathway	Low (poor bioavailability)	Human + animal (heterogeneous)	Enhanced autophagy, reduced protein aggregation, mixed cognitive outcomes
Anthocyanins	Berries	BDNF modulation	CREB–BDNF pathway	Moderate (metabolite-dependent)	Human + animal	Improved synaptic plasticity, memory, and cognitive function

Abbreviations: SIRT1, sirtuin 1; AMPK, AMP-activated protein kinase; Nrf2, nuclear factor erythroid 2–related factor 2; ARE, antioxidant response element; NF-κB, nuclear factor kappa B; mTOR, mechanistic target of rapamycin; BDNF, brain-derived neurotrophic factor; CREB, cAMP response element-binding protein; BBB, blood–brain barrier; RCTs, randomized controlled trials.

**Table 3:** Microbiota-derived polyphenol metabolites and their neuroprotective mechanisms.

Microbial Metabolite	Polyphenol Precursors	Major Microbial Transformation	Reported Biological Effects
Phenolic acids (e.g., phenylpropionic and phenylacetic acids)	Flavonoids, phenolic acids	Ring cleavage and reduction	Antioxidant activity, modulation of redox homeostasis
Phenyl-γ-valerolactones	Flavan-3-ols (catechins, epicatechins)	Microbial degradation in the colon	Anti-inflammatory effects, vascular and neuronal protection
Urolithins	Ellagitannins (pomegranate, berries, nuts)	Microbial conversion of ellagic acid	Activation of mitophagy, improved mitochondrial function
Dihydroresveratrol	Resveratrol	Microbial reduction	Anti-inflammatory activity and metabolic regulation
Enterolignans (enterolactone, enterodiol)	Dietary lignans (flaxseed, sesame)	Microbial dehydroxylation and demethylation	Hormone-like activity and antioxidant effects

**Table 4:** Comparison of polyphenol exposure levels and dose–response considerations.

Exposure Level	Typical Dose Range	Source	Bioavailability Characteristics	Relevance for Dose–Response Interpretation
Habitual dietary intake	~100–1000 mg/day (total polyphenols)	Fruits, vegetables, tea, coffee, wine	Low, variable; strong matrix and microbiome influence	Reflects real-life exposure; effects modest but physiologically relevant
Enhanced dietary intake	~500–2000 mg/day	Polyphenol-rich diets (e.g., Mediterranean diet, berries, cocoa)	Still food-matrix dependent; moderate increase in metabolites	Allows evaluation of diet-based interventions
Supplemental intake	~100–1000 mg/day (single compounds, e.g., resveratrol, EGCG)	Dietary supplements, extracts	Higher systemic exposure; altered metabolism	May exceed physiological range; relevance to diet unclear
Pharmacological/experimental doses	Often >> 1000 mg/day (or high µM in vitro)	Preclinical models	Not representative of human exposure	Limits translational relevance

Source: Authors’ own synthesis based on the reviewed literature [250,251,252,253,254,255,256].

**Table 5:** Key research gaps and priorities for clinical translation of dietary polyphenols.

Research Domain	Key Limitation	Priority for Future Research	Translational Relevance
Bioavailability	Low and variable systemic exposure	Development of novel delivery systems (e.g., encapsulation, nanoformulations)	Improve clinical efficacy
Dose–response relationships	Poorly defined, non-linear effects	Standardized dose–response studies across dietary and supplemental ranges	Establish evidence-based recommendations
Clinical trials	Small sample sizes, short duration, heterogeneity	Large-scale, long-term randomized controlled trials with standardized endpoints	Strengthen causal inference
Interindividual variability	Microbiome, genetics, metabolism differences	Precision nutrition and stratified intervention approaches	Identify responders vs. non-responders
Polyphenol interactions	Limited data on synergistic or additive effects	Investigation of combinations with other bioactive compounds	Optimize therapeutic potential
Safety and toxicity	Limited long-term human data, especially for supplements	Dose-dependent safety assessment and toxicological profiling	Ensure safe clinical application
Biomarkers	Lack of validated exposure and response markers	Integration of metabolomics-based biomarkers	Improve exposure assessment
Gut–brain axis	Mechanistic pathways not fully validated in humans	Human studies on microbiota-derived metabolites	Enhance mechanistic understanding

Source: Authors’ own synthesis based on the reviewed literature [6,14,27,30,31,32,34,35,37,85,127,128,129,141,144,172,188,228,247].

## Data Availability

Data sharing is not applicable to this article as no new data were created or analyzed in this study.

## Abbreviations

AD	Alzheimer’s disease
PD	Parkinson’s disease
ROS	Reactive oxygen species
RNS	Reactive nitrogen species
Nrf2	Nuclear factor erythroid 2–related factor 2
NF-κB	Nuclear factor kappa B
AMPK	AMP-activated protein kinase
mTOR	Mechanistic target of rapamycin
SIRT1	Sirtuin 1
BDNF	Brain-derived neurotrophic factor
CREB	cAMP response element-binding protein
BBB	Blood–brain barrier
CNS	Central nervous system
IL-6	Interleukin-6
TNF-α	Tumor necrosis factor alpha
CRP	C-reactive protein
ATP	Adenosine triphosphate
mtDNA	Mitochondrial DNA
SASP	Senescence-associated secretory phenotype
EGCG	Epigallocatechin gallate
MCI	Mild cognitive impairment
APP	Amyloid precursor protein
Aβ	Amyloid-beta
GSK-3β	Glycogen synthase kinase 3 beta
LPS	Lipopolysaccharide
PGC-1α	Peroxisome proliferator-activated receptor gamma coactivator 1-alpha
UGT	UDP-glucuronosyltransferase
SULT	Sulfotransferase
COMT	Catechol-O-methyltransferase
